# Ibuprofen-loaded electrospun poly(ethylene-*co*-vinyl alcohol) nanofibers for wound dressing applications†

**DOI:** 10.1039/d3na00102d

**Published:** 2023-03-07

**Authors:** Jean Schoeller, Karin Wuertz-Kozak, Stephen J. Ferguson, Markus Rottmar, Jonathan Avaro, Yvonne Elbs-Glatz, Michael Chung, René M. Rossi

**Affiliations:** a Empa, Swiss Federal Laboratories for Materials Science and Technology, Laboratory for Biomimetic Membranes and Textiles 9014 St. Gallen Switzerland rene.rossi@empa.ch; b ETH Zürich, Institute for Biomechanics 8093 Zürich Switzerland; c Rochester Institute of Technology (RIT), Department of Biomedical Engineering Rochester NY 14623 USA; d Empa, Swiss Federal Laboratories for Materials Science and Technology, Laboratory for Biointerfaces 9014 St. Gallen Switzerland; e Empa, Swiss Federal Laboratories for Materials Science and Technology, Center for X-ray Analytics 8600 Dübendorf Switzerland; f School of Engineering, The University of Edinburgh King's Buildings EH9 3JL Edinburgh UK

## Abstract

Chronic wounds are characterized by a prolonged inflammation phase preventing the normal processes of wound healing and natural regeneration of the skin. To tackle this issue, electrospun nanofibers, inherently possessing a high surface-to-volume ratio and high porosity, are promising candidates for the design of anti-inflammatory drug delivery systems. In this study, we evaluated the ability of poly(ethylene-*co*-vinyl alcohol) nanofibers of various chemical compositions to release ibuprofen for the potential treatment of chronic wounds. First, the electrospinning of poly(ethylene-*co*-vinyl alcohol) copolymers with different ethylene contents (32, 38 and 44 mol%) was optimized in DMSO. The morphology and surface properties of the membranes were investigated *via* state-of-the-art techniques and the influence of the ethylene content on the mechanical and thermal properties of each membrane was evaluated. Furthermore, the release kinetics of ibuprofen from the nanofibers in a physiological temperature range revealed that more ibuprofen was released at 37.5 °C than at 25 °C regardless of the ethylene content. Additionally, at 25 °C less drug was released when the ethylene content of the membranes increased. Finally, the scaffolds showed no cytotoxicity to normal human fibroblasts collectively paving the way for the design of electrospun based patches for the treatment of chronic wounds.

## Abstract 2

This work presents the production of poly(ethylene-*co*-vinyl alcohol) nanofibers by electrospinning and the capacity of the fibers to act as a patch loaded with ibuprofen for wound healing applications is evaluated.

## Graphical abstract

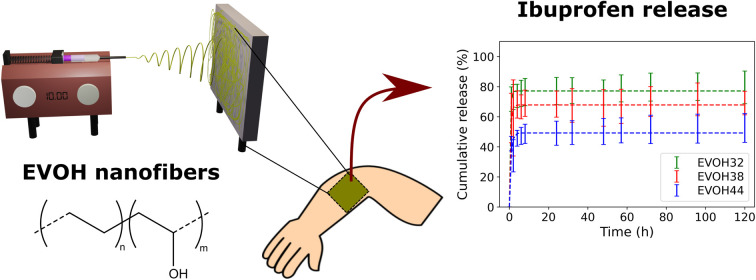

## 1 Introduction

Wound healing is a complex phenomenon involving a cascade of steps starting with inflammation as a healthy part of the process.^1–3^ However, in chronic wound healing, a prolonged inflammation phase disrupts the normal healing mechanism.^4,5^ Such wounds are known to cause severe distress for the patient and be a significant financial burden for the healthcare system.^6^ For this purpose, non-steroidal anti-inflammatory agents (NSAIDs), in particular ibuprofen (IBU), have emerged as a potential solution to reduce inflammation and relieve pain in chronic wound healing. The development of NSAID-loaded topical wound dressings has been shown to promote healing of chronic wounds as well as locally reducing pain for the patients.^7,8^ Furthermore, skin temperature during wound healing fluctuates between 30 and 34 °C, where higher temperatures have been associated with impaired healing.^9,10^ Therefore, the development of thermoresponsive vectors for the delivery of IBU, when skin temperature exceeds the normal range, could be innovative for the treatment of chronic wounds. In fact, Andgrie *et al.* have designed an injectable hydrogel to locally administer NSAID, but more porous and breathable solutions are desired.^7^

For this purpose, electrospinning is a robust and well-established method for the fabrication of mechanically stable meshes composed of nanofibers (NFs) with diameters on the nanoscale.^11^ Indeed, by applying an electrical field to a polymer solution emitted at a given flow rate, a jet is formed and while the jet travels to a collector, the solvent evaporates leading to the deposition of solid nanofibers. This technique leads to the production of highly porous nanofibrous membranes exhibiting one of the highest surface-to-volume ratios in materials sciences.^12^ Consequently, electrospun NFs have been in the spotlight for the design of drug delivery systems in the past few decades due to their aforementioned inherent properties and the ability to mix pharmaceutical agents within the polymeric solution used for electrospinning and direct drug encapsulation within the NFs.^13–17^ As the scaffolds inherit the physical properties of the polymer used for electrospinning, the choice of the polymer system allows for specific designs of membranes able to respond to changes in physiological and environmental conditions (pH, temperature, light, *etc.*).^18,19^ These unique properties have motivated the development of electrospun-based wound dressings in the past few decades.^20–24^ For example, Morgado *et al.* have developed a PVA/chitosan patch able to release IBU over 3 days whereby *in vivo* efficiency testing showed that the wounds healed faster when IBU was loaded into the scaffolds.^8^

**Fig. 1:**
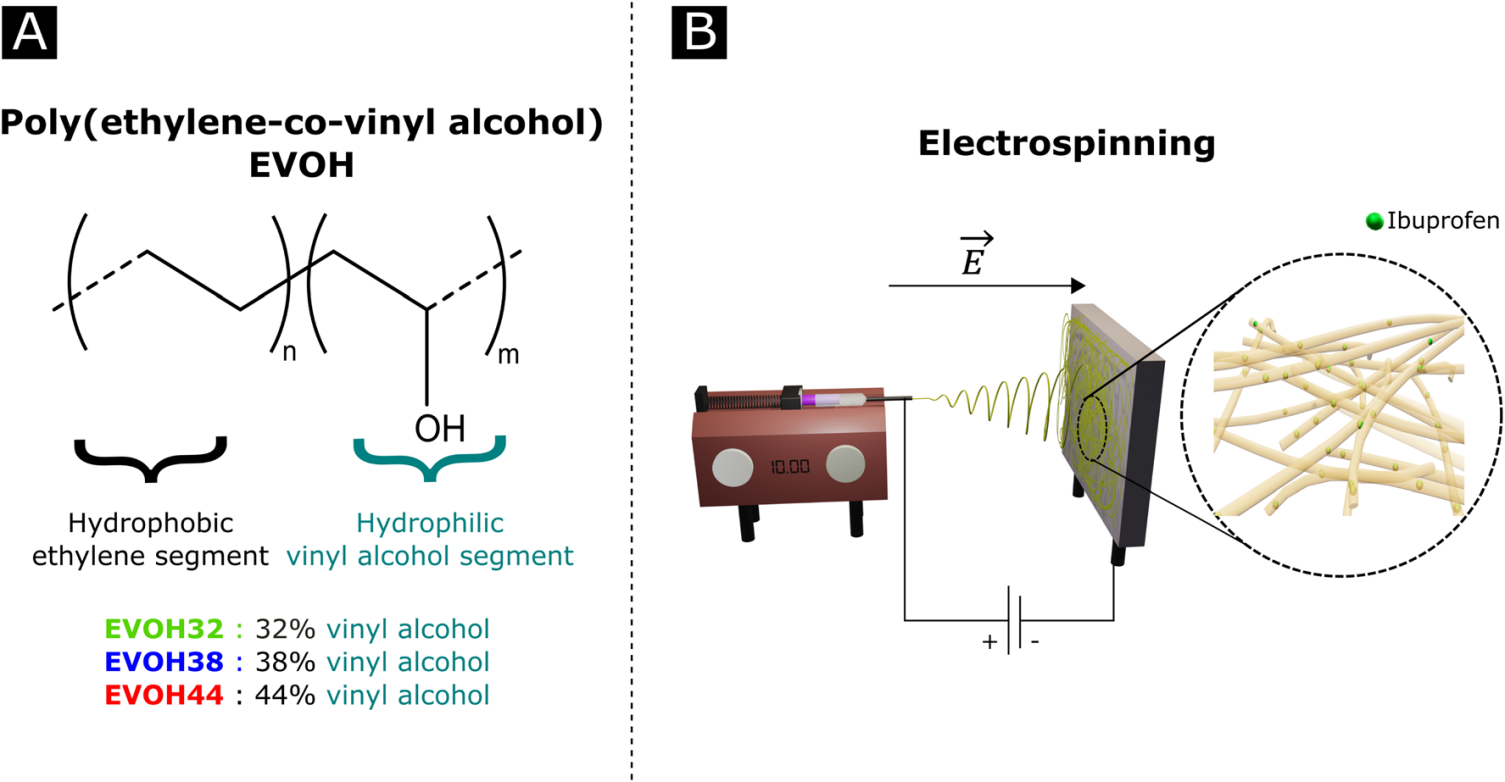
(A) Poly(ethylene-*co*-vinyl alcohol) (EVOH) copolymers used in this study and (B) a graphical representation of an electrospinning setup.

Poly(ethylene-*co*-vinyl alcohol) (EVOH, Fig. 1) is a copolymer of poly(ethylene) (PE) and poly(vinyl alcohol) (PVA) derived from the hydrolysis of poly(ethylene-*co*-acetate), with a glass transition temperature close to physiological temperature, making it a promising candidate for temperature-responsive applications. While EVOH has been mainly used for food packaging in the past few decades, due to its excellent gas barrier properties, this material receives growing interest for thermosensitive drug delivery systems.^25–27^ Particularly, Hassanzadeh *et al.* have reported the fabrication of folic acid-targeted succinylated EVOH nanoparticles and demonstrated a higher release of epirubicin as a function of temperature.^28^ The release kinetics were explained by the structural degradation of EVOH at higher temperatures, leading to the release of epirubicin, thus revealing the critical impact of nanoscale structural changes for drug release application of this material. Furthermore, by adjusting the ratio of vinyl alcohol to ethylene segments, the thermal and mechanical properties of the copolymer can be finely tuned for the desired purpose.

The fabrication of EVOH electrospun NFs has been previously described in several solvent systems, the most common being a mixture of isopropanol and water, resulting in a broad fibre diameter distribution with average diameters ranging from 0.6 to 2 μm.^29–31^ Unfortunately, such solutions are known to rapidly precipitate at room temperature, thus hindering the electrospinning process once crystalline forms are present.^30^ Other reported solvent systems are hexafluoro-2-isopropanol (HFIP) and HFIP/water mixtures,^29,32^ which however suffer from inherent limitations in the biomedical domain due to the toxicity of HFIP. Furthermore, the current state of research and market demands require the development of greener solvents to minimize the environmental impact of electrospinning.^33^ While dimethyl sulphoxide (DMSO) may act as a greener alternative, its use for the electrospinning of EVOH is poorly reported in the literature.^34,35^

In an effort to develop a IBU loaded electrospun patch for chronic wound healing (Fig. 1), we optimized the electrospinning of EVOH copolymers with different ethylene contents (32, 38 and 44 mol%) in DMSO by adjusting both the solution and process properties. The membranes were then characterized in terms of *ζ*-potential, water contact angle and chemical composition *via* Fourier-transform infrared spectroscopy, X-ray scattering and X-ray photoelectron spectroscopy. The thermal and mechanical behaviors were investigated to assess the ability of EVOH nanofibers to be used as a wound-healing patch. Moreover, IBU was encapsulated within the nanofibrous scaffolds for different ethylene contents and its release kinetics were evaluated at 25 °C and 37.5 °C. Finally, the cytocompatibility of the scaffolds was assessed with normal human fibroblasts.

## 2 Materials and methods

### Materials

Poly(ethylene-*co*-vinyl alcohol) (EVOH) with 44 mol% (EVOH44, Soarnol A4412) and 38 mol% (EVOH38, Soarnol A3808) ethylene content was purchased from Nippon Gohsei (Nippon Gohsei, Japan) and used without further modifications. EVOH with 32 mol% ethylene content (EVOH32), benzyltriethylammonium chloride (BTEAC), dimethyl sulfoxide (DMSO), ibuprofen (IBU) and phosphate buffered saline (PBS) tablets were bought from Sigma Aldrich (Sigma Aldrich, Switzerland) and used as received.

### Electrospinning

The electrospinning of EVOH was performed using a previously reported custom-made electrospinning apparatus.^36^ To optimize the electrospinning conditions, three different EVOH copolymers (EVOHA44, EVOH38 and EVOH32) were dissolved in 100% DMSO at a concentration of 6, 8, 10, 12, 14, 16, 18, 20, and 22 (w/w)%. The solutions were shaken at 50 °C until the polymer was completely dissolved and after cooling down, the solutions were electrospun under 4 different conditions depicted in Table 1.

**Table 1:** Parameters used for the electrospinning of EVOH nanofibers

Potential difference (kV)	Distance (cm)	Flow rate (μL min^−1^)
10/−5	10	10
10/−5	10	20
15/−5	20	10
15/−5	20	20

Once the concentration, syringe-collector distance and flow rate were optimized, the voltage was fine-tuned to obtain a stable jet allowing for long electrospinning times. Electrospun membranes were then fabricated by spinning 2 mL of the solution onto a rotating drum collector (50 rpm) covered in aluminum foil. The electrospun membranes were then detached from the aluminum foil and left to dry at 40 °C for 72 h in a vacuum oven. To prepare drug-loaded membranes, IBU was directly dissolved at a concentration of 10 (w/w)% towards the weight of EVOH inside the polymer solution.

### X-ray photoelectron spectroscopy

X-ray photoelectron spectroscopy (XPS) measurements were performed using a Quantum 2000 X-ray photoelectron spectrometer (Physical Electronics, Minnesota, United States), equipped with an Al Kα monochromatic source. Survey and high-resolution spectra were acquired at a pass energy of 117.4 eV (energy step of 1 eV) and 29.35 eV (energy step of 0.125 eV), respectively. The atomic fractions *x*_a_ of the elements were calculated using eqn (1):1
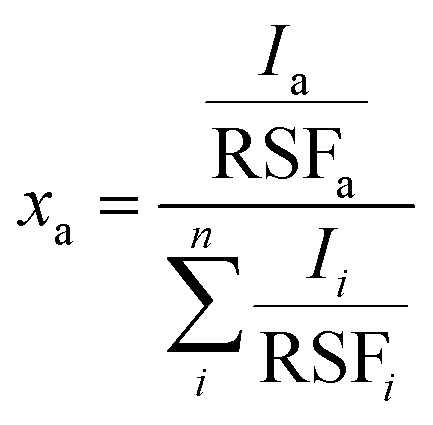
where the relative sensitivity factors (RSF) were obtained from XPS software (Multipak©) and Tougaard backgrounds were subtracted to measure the peak intensity (*I*_*i*_).

### Wide-angle X-ray scattering

Transmission wide-angle X-ray scattering (WAXS) of nanofibrous samples was performed on a Bruker NanoStar (Bruker AXS GmbH, Karlsruhe) where the instrument was equipped with a pinhole collimation system allowing a beam size at a sample position of about 400 μm in diameter. X-ray generation was sustained with a micro-focused X-ray Cu source (wavelength Cu Kα = 1.5406 Å), and scattering patterns were recorded on a 2D MikroGap technology-based detector (VANTEC-2000 2D with 2048 × 2048 pixels and 68 × 68 μm each pixel size) along with a custom-built semi-transparent beam stop. The sample-to-detector distance was set at 5 cm and further calibrated with a corundum powder standard. The scattering patterns were recorded at room temperature under moderate vacuum conditions (10^−2^ mbar) to limit air scattering. The intensity of the semi-transparent beamstop from direct beam scans was used for transmission normalization. Background subtraction using air scattering was done systematically for all samples by normalizing the scattering intensity of the direct beam and subtracting the contribution from the background to the normalized samples.

### *ζ*-Potential measurement

The surface *ζ*-potential of the electrospun meshes was measured from pH 3 to 8, using a Surpass 3 electro-kinetic flow through a *ζ*-potential meter (Anton Paar, Graz, Austria). The membranes were first rinsed 3 times with the electrolyte (1 mM KCl) solution at the respective pH prior to the measurement. The *ζ*-potential was then derived from the following equation:2
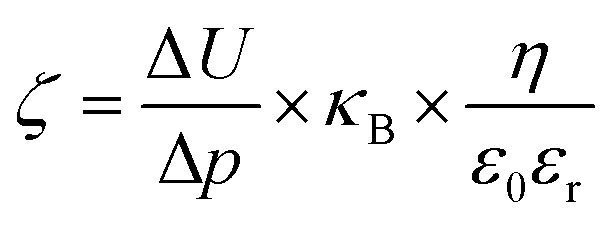
where Δ*p* is the pressure difference (kg ms^−2^), *κ*_B_ the conductivity of the solution (S m^−1^), *η* the viscosity (kg ms^−1^), *ε*_r_ the dielectric constant and *ε*_0_ is the permittivity (F m^−1^).

### Water contact angle

To measure the contact angle of the electrospun NFs, a 2 μL milliQ water droplet was deposited onto the surface of the scaffold at 20 °C using a drop shape analyzer (Krüss GmbH, Germany). The contact angle was then calculated from pictures of the droplet taken over time with an optical camera using the associated software (Advance, Krüss GmbH, Germany). The measurements were repeated three times on each fiber sample.

### Thermal analysis

TGA measurements were performed on a NETZCH TG209 F1 Iris instrument (NETZSCH, Germany) to study the thermal degradation of the different membranes. Samples of 5 mg were placed in an 85 μL alumina crucible and heated from 25 to 800 °C at a heating rate of 10 °C min^−1^. The measurements were performed under a nitrogen atmosphere to prevent any possible thermo-oxidative degradation.

DSC curves were recorded on a NETZCH DSC 214 Polyma (NETZSCH, Germany) under a nitrogen atmosphere at a heating rate of 10 °C min^−1^ to measure the typical transitions (glass transition, melting temperature, or recrystallization temperature). The specimens of 5–7 mg were compressed to the bottom of an aluminum crucible with a pierced lid and heated from 25 °C to 300 °C and then cooled down to −40 °C and heated again to 300 °C. The typical thermal transitions and weight loss were measured using the associated software (NETZCH Proteus Thermal Analysis Version 7.1.0). The degree of crystallinity of the different specimens was calculated using eqn (3):3
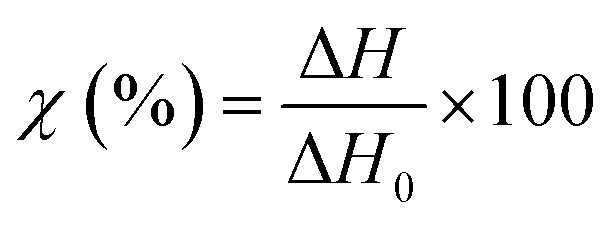
where *χ* is the degree of crystallinity, Δ*H* is the enthalpy of melting and Δ*H*_0_ is the enthalpy for melting of the pure polymer calculated according to eqn (4):

4 Δ*H*_0_ = *x*_PE_Δ*H*^PE^ + *x*_PVA_Δ*H*^PVA^

where *x*_PE_ and *x*_PVA_ are the molar fraction of ethylene and vinyl alcohol within the copolymer, and Δ*H*^PE^ and Δ*H*^PVA^ are the enthalpy of melting of 100% crystalline PVA and PE, respectively.^37^

### Mechanical testing

For mechanical testing, sample dimensions were based on the dog bone structure (specimen type 2) outlined in ISO 527-3:2018 with a gauge length of 15 mm, a width of 4 mm, and thicknesses of 59.5 ± 14.3 μm.

Tensile tests were carried out on a Zwick Z100 Universal Tester (Zwick, Germany) equipped with a 10 N load cell. The distance between the pneumatic clamps was set to 60 mm and the strain rate at 10 mm min^−1^ using testXpert II software (Zwick, Germany).

Quasi-static cyclic tensile testing was conducted using an Instron 3369 Universal Testing System (Instron, UK) with a 1 kN load cell. 25 × 25 mm^2^ clamps were used to secure samples, with a single degree of freedom along the *y*-axis, and 50 tensile cycles up to a maximum strain of 30% were applied to each sample at a strain rate of 50 mm min^−1^.

### Drug release

The relative release of IBU was evaluated at 25 °C and 37.5 °C in phosphate-buffered saline (PBS) solution. For this purpose, drug-loaded membranes were cut into strips (4.96 ± 1.56 mg) and immersed in 3 mL of PBS. At specific time points (1, 2, 4, 6, 8, 24, 32, 48, 57, 72,96 and 120 h), 300 μL of the supernatant were collected and replaced with 300 μL of fresh PBS to keep the volume constant at 3 mL. The supernatants were diluted to 500 μL and quantified using ultra-high-performance liquid chromatography (Acquity UHPLC, Waters Inc., Baden, Switzerland) equipped with a UV-vis detector as previously reported.^38^ Separation was achieved by reverse-phase gradient elution from 95/5% to 5/95% (v/v) water/acetonitrile over 5 min at 40 °C. The mobile phase was delivered at a flow rate of 0.5 mL min^−1^ through an Acquity® C18 column (2.1 × 50 mm, 1.7 μm). The detector wavelength was set at 221 nm, and the injection volume at 15 μL. Calibration curves using standard concentrations of IBU were recorded and used for quantification using the MassLinx® interface. The integration parameters and the calibration curve for the experiments can be found in Fig. S4† as well as Table S1.†

The release curves were fitted with a desorption model, previously reported by Srikar *et al.*, according to eqn (5):^39^5
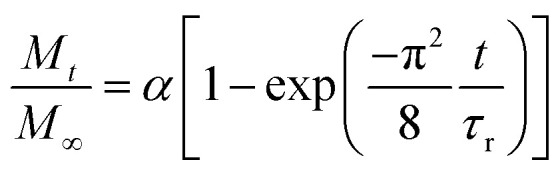
where *M*_*t*_ is the mass of drug released at time *t*, *M*_*∞*_ is the maximum amount of drug possibly released, *α* is the nanoporosity factor, and *τ*_r_ is the characteristic time.

### Cytocompatibility assessment

The cytocompatibility of drug-loaded as well as plain electrospun fiber membranes was evaluated using normal human dermal fibroblasts (NHDF-c adult (47 year old) (Promocell, Switzerland, Lot 410Z037.5)). For this, material extracts were prepared by incubating the scaffolds (21.5 ± 3.5 mg) in Dulbecco's Modified Eagle Medium (DMEM) at a weight to volume ratio of 0.01 mg mL^−1^ for 24 h at 37.5 °C on a shaker at 300 rpm. NHDFs were seeded into tissue culture multi-well plates at a density of 5000 cells per cm^2^ in proliferation medium (DMEM supplemented with 10% fetal calf serum (FCS), 2% glutamine and 1% penicillin/streptomycin) and cultured overnight before replacing the proliferation medium with material extracts (supplemented with 10% FCS, 2% glutamine and 1% penicillin/streptomycin). NHDFs were then cultured at 37.5 °C for 24 h and 72 h before assessing cell viability using a metabolic activity assay (alamarBlue Cell Viability Reagent, Thermo Fisher, Switzerland) and total DNA content (Quant-iT PicoGreen dsDNA Reagent and Kits, Thermo Fisher, Switzerland).

### Statistical analysis

Results are displayed as mean ± standard deviation. Statistical significance was assessed by a Kruskal–Wallis test for the groups followed by pairwise comparison with the Dunn test (Bonferroni corrections), and the results were accepted as significantly different for *p* < 0.05. All statistical analysis was performed using R.^40^

## 3 Results and discussion

Due to the reported precipitation of EVOH in IPA/H_2_O mixtures after a few hours and the toxicity of HFIP-based solvent systems, the suitability of DMSO as a solvent for the electrospinning of EVOH was explored.^30,41^ Although DMSO possesses a high boiling point (189 °C), it is non-toxic at low concentrations and suitable for the design of biomedical scaffolds.^42,43^ Additionally, EVOH solutions prepared with DMSO were stable over time and no precipitation was observed after several weeks. For all three copolymers (EVOH32, EVOH38 and EVOH44), concentrations from 6 to 12 (w/w)% led to poor electrospinning with a combination of electrospinning and electrospraying for all the conditions depicted in Table 1. From a concentration of 12 up to 22 (w/w)%, electrospinning was the predominant phenomenon over electrospraying. The SEM images for all concentrations are available in the ESI (Fig. S1–S3†).

**Fig. 2:**
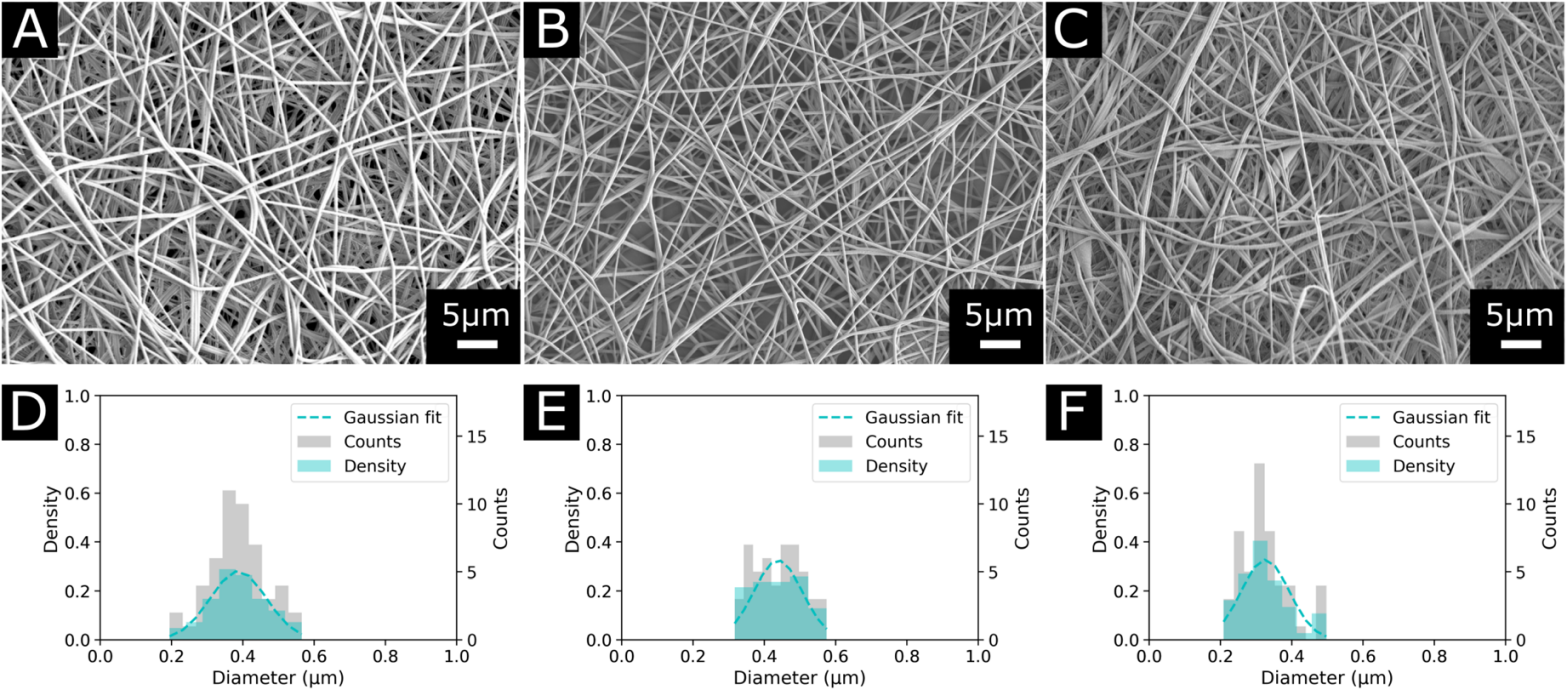
Morphology and diameter distribution of the final electrospun nanofibers used in this study for each copolymer (A) and (D) EVOH44, (B) and (E) EVOH38 and (C) and (F) EVOH32.

We found that electrospinning EVOH38 and EVOH44 was uninterrupted and produced beadless NFs at a concentration of 20 (w/w)%, while for EVOH32 a lower concentration of 18 (w/w)% produced similar results. To lower the diameter of the fibers, BTEAC salt was added to the solution at a concentration of 0.01 (w/w)% towards the polymer weight to increase the conductivity of the polymer solution, which has been previously shown to decrease the fiber diameter.^44^ This led to the formation of NFs on the nanoscale with an average diameter of 325 ± 67, 387 ± 79, and 438 ± 68 nm for EVOH32, EVOH38 and EVOH44 respectively (Fig. 2). Due to the low vapor pressure and high boiling point of DMSO some droplets occasionally occurred, which however did not affect the electrospinning process. Furthermore, the addition of IBU to the electrospinning solution did not affect the jet and produced fibers of similar diameters.

**Fig. 3:**
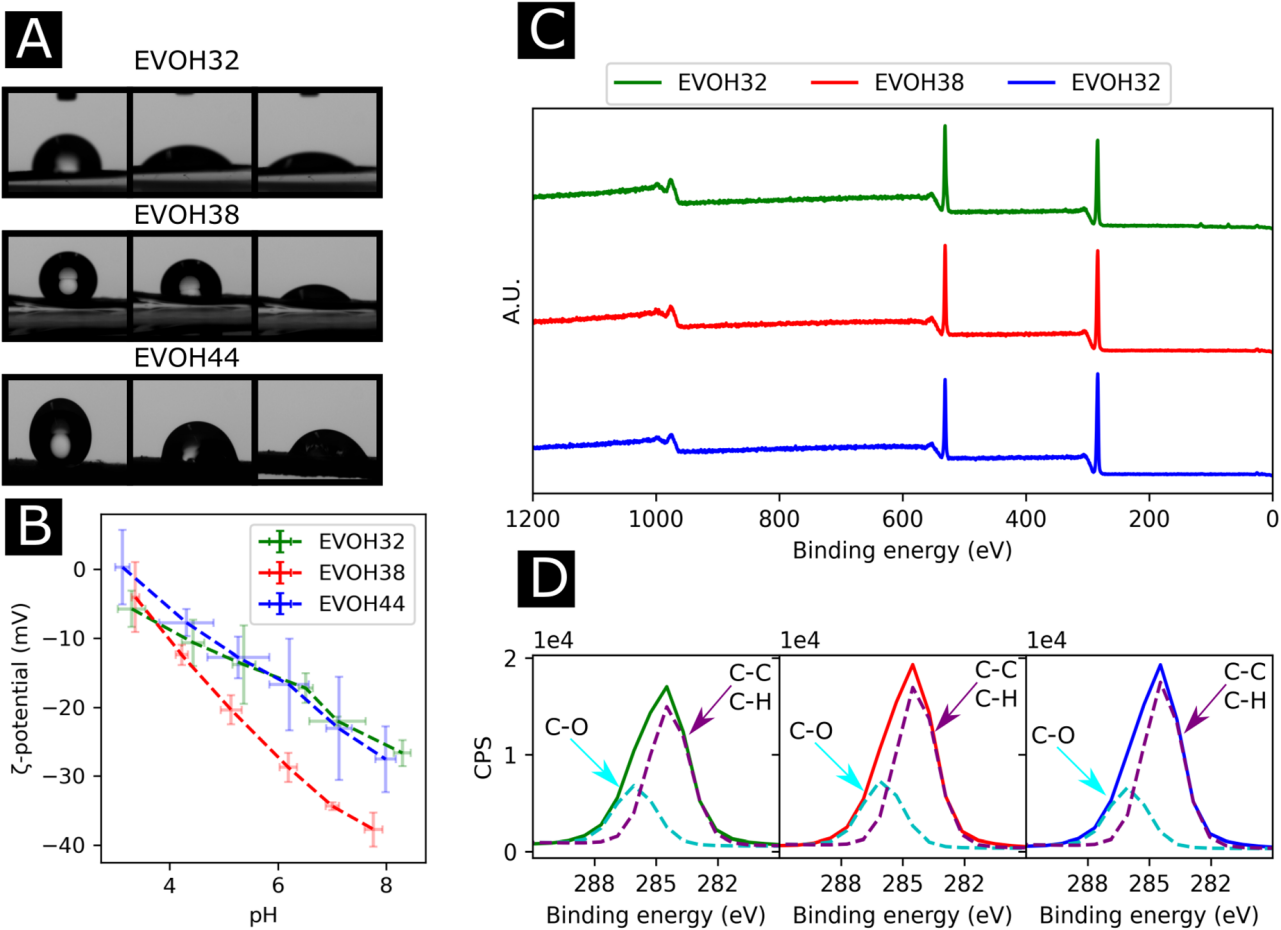
(A) Images of the sessile drop measurement at 1 s, 30 s and 90 s (from left to right), (B) surface *ζ*-potential from pH 3 to pH 9, (C) X-ray photoelectron spectroscopy survey scans of the membranes and (D) region scan for the C 1s environment for each EVOH copolymer.

To understand the wetting properties of the EVOH membranes, sessile drop measurements were performed (Fig. 3(A)). The measured contact angle just after depositing the drop (1 s) varied from 93.0 ± 11.8°, 116.35 ± 14.2° to 126.8 ± 7.6° for EVOH32, EVOH38 and EVOH44, respectively. This increase in the contact angle can be attributed to the smaller number of –OH groups with increasing ethylene content, leading to more hydrophobic membranes. However, after 30 seconds these values dropped to 54.3 ± 7.3°, 90.9 ± 19.9° and 87.7 ± 2.8° and after 90 seconds to 49.1 ± 8.8°, 55.8 ± 14.1° and 58.8 ± 4.2° due to the transition from a Cassie–Baxter state to a Wenzel state. Indeed, when a drop is deposited on porous samples such as electrospun meshes, where air is trapped underneath, an increase of the contact angle compared to the pristine bulk material can be observed due to the Cassie–Baxter nature of the interaction.^45,46^ After depositing the drop, the wetting state quickly transitioned towards a Wenzel state to minimize the surface energy by spreading throughout the membrane's pores.^47^ This demonstrates that although EVOH has been reported to be a hydrophobic material, the processing into nanofibrous mats led to a decrease in the contact angle and higher hydrophilicity, which are desired for wound dressings to keep the environment moist to promote healing.

To further elucidate the surface energy and composition of the scaffolds, *ζ*-potential and XPS measurements were performed (Fig. 3(B) and (C)). The *ζ* potential of the copolymer was slightly negative from pH 3 to pH 8 further decreasing with increasing pH. This is explained by the –OH groups being deprotonated with increasing pH, thus reducing the surface charge.^48^ No changes were observed when varying the ethylene content except for EVOH38, which showed lower values over the whole pH range. This was unexpected as a higher molar fraction of –OH groups should have led to lower surface *ζ*-potential values. Furthermore, the XPS survey scans revealed a surface atomic concentration of carbon of 71.4, 75.8 and 77.49% while the oxygen concentration was 28.6, 24.2 and 22.51% for EVOH32, EVOH38 and EVOH44, respectively. As expected, the concentration of carbon atoms at the surface was lower when decreasing the ethylene content.^49^ Furthermore, the region scan of the carbon environment revealed a higher proportion of C–O bonds when the ethylene content decreased. The typical bands for EVOH were also observed in FTIR and detailed in the ESI (Fig. S5†). These results confirm the chemistry of the polymeric backbone of the three copolymers used in this study.

**Fig. 4:**
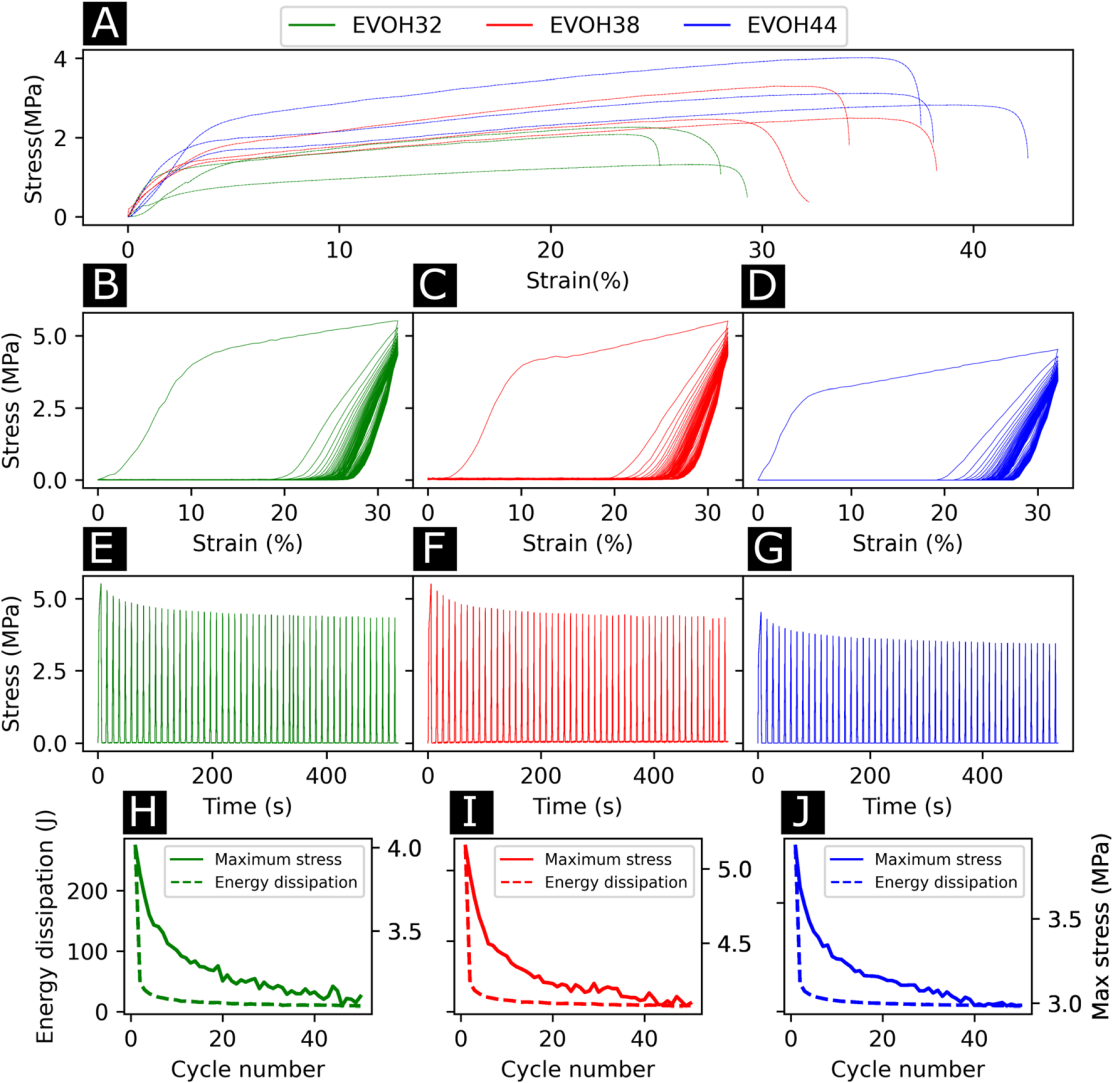
(A) Stress–strain curves for the three EVOH electrospun membranes. Stress over strain of quasi-static cyclic tensile testing for 50 cycles for (B) EVOH32, (C) EVOH38 and (D) EVOH44 (average of three independent samples). Stress over time for (E) EVOH32, (F) EVOH38 and (G) EVOH44 for 50 cycles (examples of single specimens). Energy dissipation and maximum stress for (H) EVOH32, (I) EVOH38 and (J) EVOH44 for each cycle (average of three independent samples).

To study the effect of increasing ethylene content on the mechanical properties of the membranes, tensile testing experiments were performed on each of the copolymers (Fig. 4(A)). Young's moduli of 27.6 ± 9.0, 32.5 ± 4.5, 54.2 ± 10.3 MPa were derived from the stress–strain curves for EVOH32, EVOH38 and EVOH44, respectively, revealing that a lower ethylene content within the copolymer led to consistent reduction in the modulus, which can be explained by the higher compliance of the vinyl alcohol component of the copolymer in comparison to the stiffer polyethylene segment.^50,51^ The extension at break was found to be 28.1 ± 1.6, 34.1 ± 6.8 and 37.7 ± 2.0% for EVOH32, EVOH38 and EVOH44 demonstrating that, even though the increase in polyethylene led to stiffer membranes, the membranes with a higher ethylene content could sustain higher strains.

Throughout their lifetimes, the EVOH patches will likely experience continuous mechanical stress and understanding how the membranes respond to such stress is important for future applications as wound dressings. Therefore, we investigated the mechanical stability of the three EVOH membranes using quasi-static cyclic tensile testing up to 30% strain over 50 cycles. When applying quasi-static cyclic strain, the maximum strain remained unchanged but the loading curve consistently displayed higher mechanical stress compared to the unloading curve of the cycle (Fig. 4(B)–(D)). The large areas between the loading/unloading curves demonstrate high hysteresis behavior of the membranes with energy dissipated as heat. This is due to the non-linear viscoelastic behavior of the samples under stress where the energy dissipation derives from the internal friction of polymer chains and at higher strains, the breakage of fiber-to-fiber interactions. The stress–time (Fig. 4(E)–(G)) curves show the evolution of stress over time where it can be seen that the maximum stress decreased throughout the cycles. This is also depicted in Fig. 4(H)–(J), where an exponential decrease of the maximum stress can be observed with increasing cycle number for the three copolymers. This is referred to as the Mullins effect or stress softening, where the maximum stress decreases after each loading cycle, thus representing the typical aforementioned non-linear elastic behavior.^52^ Interestingly, EVOH44 maximum stress throughout the cycles was lower when compared to EVOH32 and EVOH38. This is explained by the fact that reducing the number of vinyl alcohol segments decreased the maximum stress due to less intramolecular hydrogen bonding emerging from the –OH bonds. To visualize the hysteresis of the material throughout the cycles the energy dissipation was plotted alongside the maximum stress (Fig. 4(H)–(J)). A steep decrease in the energy dissipation can be observed due to the mesoscopic changes during the tensile stress as well as the shear within the electrospun membranes leading to fiber-to-fiber interaction disruption.^53^ Furthermore, a major reduction in energy dissipation was observed between cycles 1 and 2, which can be explained by the plasticization of the weaker fibers in the membrane as the applied strain is higher than the yield point of the membranes in addition to typical stress softening behavior. Overall, the mechanical stability of the membranes throughout the cyclic testing experiments demonstrates their ability to be used as wound healing patches.

**Fig. 5:**
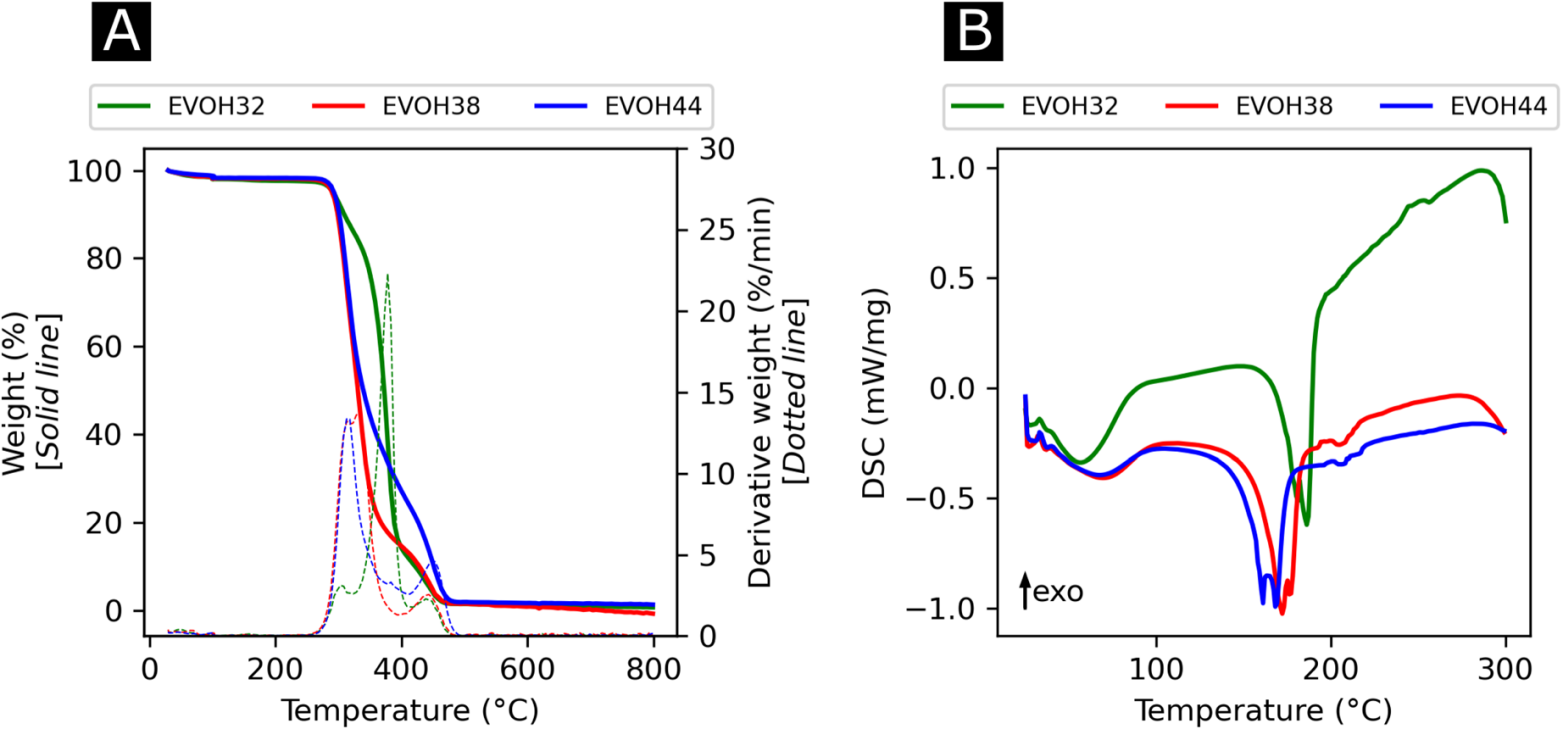
(A) Thermogravimetric analysis curves with the respective first derivatives and (B) differential thermograms of the three EVOH copolymers.

In an effort to understand the thermoresponsive behavior of EVOH copolymers, the thermal properties of the EVOH copolymers were investigated *via* TGA and DSC (Fig. 5) to determine each specimen's typical thermal transitions. In the TGA curves, the first weight loss centered at 378, 332.3 and 316 °C for EVOH32, EVOH38 and EVOH44, respectively, can be attributed to the vinyl alcohol component of the EVOH copolymers. Therefore, the weight loss was greater for a higher vinyl alcohol content (82, 78 and 66% for EVOH32, EVOH38 and EVOH44 respectively). The second weight loss centered at 447, 447.4 and 449.4 °C corresponds to the ethylene segment of EVOH. This weight loss increased with the number of ethylene units within the copolymer (13, 18 and 30% for EVOH32, EVOH38 and EVOH44, respectively). Overall, the onset of the EVOH NF degradation was high enough to consider the membranes stable for usage at ambient temperatures.

To further investigate the thermal properties of the EVOH copolymers, differential thermograms were recorded for each EVOH electrospun nanofibrous membrane. The first endothermic transition occurring at 57.5, 61.0, and 62.8 °C for EVOH32, EVOH38 and EVOH44, respectively, was attributed to the evaporation of the water absorbed by the membranes and the glass transition of the copolymers. After a second heating cycle (Fig. S6†), the endothermic peak disappeared hindering the measurement of a glass transition for the copolymers. Nevertheless, it was previously reported that a lower ethylene content increases the glass transition temperature due to the addition of the –OH group forming intramolecular hydrogen bonds that prevent the mobility of the chains.^54^ Also, pure IBU was measured and the melting temperature of 78.2 °C was confirmed (Fig. S7†).

The second endothermic transition at 186.3, 172.3 and 161.7 °C for EVOH32, EVOH38 and EVOH44 corresponds to the melting temperature of the copolymers. As PVA has a higher melting temperature than PE, it was expected that EVOH32 has a higher melting temperature. Additionally, the degree of crystallinity of the copolymers calculated using eqn (3) was 47.9, 36.2 and 34.1% for EVOH32, EVOH38 and EVOH44 respectively. This confirms the results of Rwei *et al.*, which have found a similar trend for melt-spun fibers, but contradicts the study from Alvarez *et al.*, where it was shown that crystallinity increased with increasing ethylene content.^55,56^ Further investigating the degree of crystallinity of the copolymers, WAXS data were recorded for each copolymer (Fig. S7†). Only the amorphous component could be observed on the samples and, due to the absence of the crystalline diffraction, no degree of crystallinity or diffraction spacing could be calculated with the deconvolutions. However, a slight change of the peak position of the amorphous peak was recorded suggesting a slight change of the fibers' nano-domains with increasing ethylene content.

**Fig. 6:**
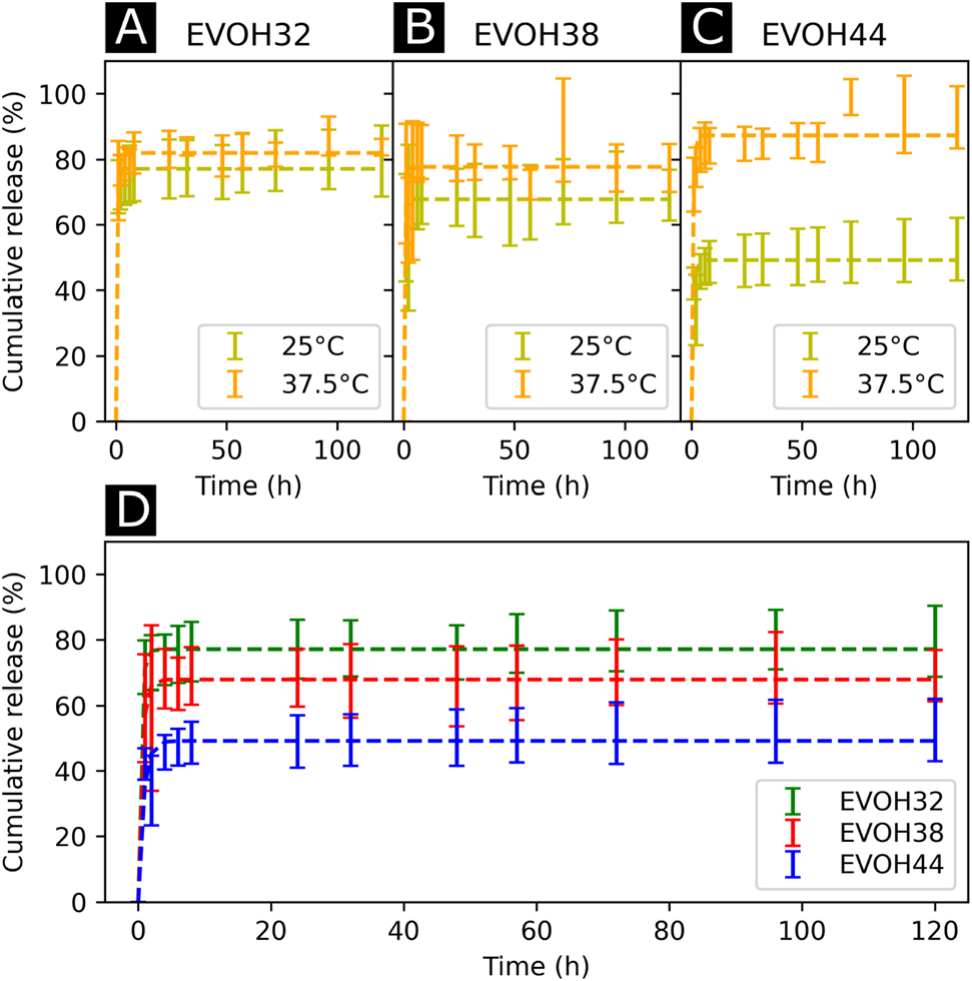
Drug release kinetics of ibuprofen at 25 °C and 37.5 °C for (A) EVOH32, (B) EVOH38 and (C) EVOH44. (D) Influence of the ethylene content on the release of ibuprofen at 25 °C. The curves were fitted with a desorption model according to eqn (5).

After assessing the mechanical and thermal performances of the patches, the release kinetics of IBU from the electrospun membranes were investigated. To study the release rates of the different patches, the membranes were immersed in PBS and shaken at different temperatures (25 and 37.5 °C, Fig. 6(A)–(C)). A burst release could be observed for all three copolymers, with most of the drug (42–73%) being released within the first 4 hours. This release profile can be explained by the higher affinity of IBU with DMSO compared to EVOH. EVOH requires a certain time and heating to dissolve in DMSO, while the quantity of IBU used in this study is highly soluble in DMSO. Therefore, as DMSO evaporates during and after electrospinning, IBU is expected to remain within the DMSO and thus migrate to the fiber surface, leading to a higher IBU concentration at the surface than in the bulk of the fibers. Therefore, the burst release observed may be explained by the desorption of the drug from the surface when immersed in PBS. This was confirmed by fitting the release curves with a desorption model (eqn (5)) showing a good fit (Table S2†) for the experimental data.^39^ Therefore, only the IBU on the fibers or pore surfaces was released, while the IBU trapped in the bulk of the fibers remained on the observed time scale. Such a fast release of ibuprofen is an attractive process for the use of emergency anti-inflammatory patches.

**Fig. 7:**
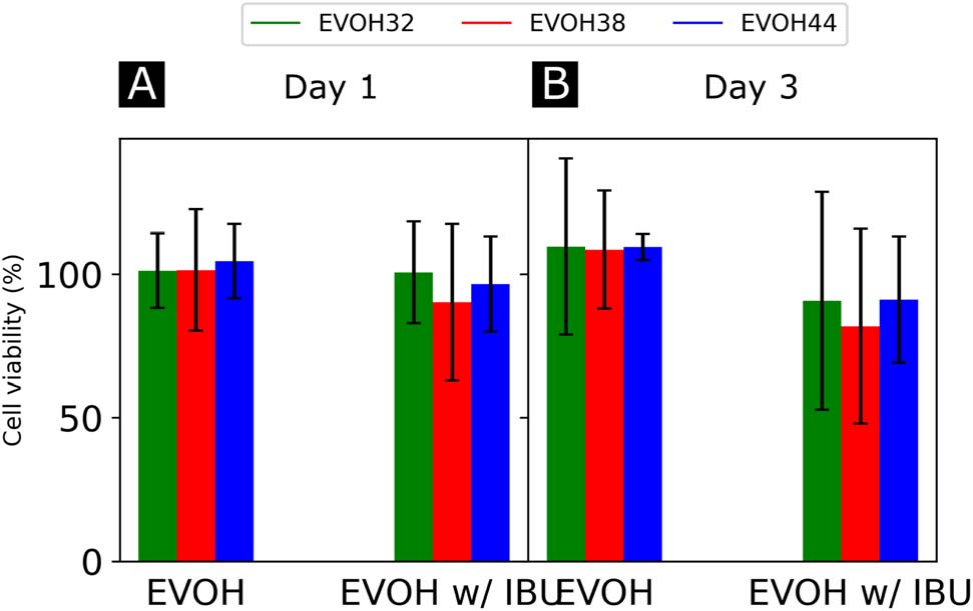
Cell viability and total DNA content of normal human fibroblasts incubated with 24 h extracts from the electrospun membranes after (A) day 1 and (B) day 3 of culture, normalized to control (culture medium), and *n* = 4.

For all EVOH copolymers, more IBU was released at 37.5 °C than at 25 °C (Fig. 7(A)–(C) and Table S3†). Nevertheless, a considerable amount of drug (71.7 ± 8.1%, 59.1 ± 16.4% and 42.1 ± 5.0% for EVOH32, EVOH38 and EVOH44, respectively) was released from the fibers at 25 °C when compared to the study of Hassanzadeh *et al.* where most of the drug remained within the nanoparticles at low temperatures.^28^ Such a difference between the release profiles could be explained by the fabrication process of the fibers leading to the aforementioned migration of IBU to the surface of the NFs. Two competing mechanisms can explain this temperature sensitivity. First, the higher solubility of IBU in PBS at 37.5 °C than at 25 °C allows for a greater amount of IBU to be released. Also, as the temperature increases and approaches the copolymers' glass transition, the higher mobility of the chains in the polymeric matrix allows for a more important release of IBU.

Additionally, the influence of the ethylene content within the electrospun membranes on the release of IBU was highlighted (Fig. 6(D)). It was found that at 25 °C, higher ethylene contents led to less IBU being released from the membranes. As IBU is hydrophobic, this slower release can be explained by strong hydrophobic interactions existing between the longer ethylene segments preventing the release of the drug. Also, negative electrostatic charge repulsion between the –OH groups of the vinyl alcohol segment and IBU favors the release of the drug thus explaining the observed trend. This hydrophobic interaction of IBU with the ethylene segment of EVOH could also lead to more drug being trapped within the polymeric matrix during its migration to the surface thus limiting the desorption mechanism previously mentioned. Interestingly, this trend evolved linearly with the ethylene content as shown in Fig. S8† and was not observed at 37.5 °C where no difference was observed between the groups.

Finally, the cytocompatibility of the electrospun membranes was evaluated with an indirect assay using material extracts (Fig. 7). When culturing NHDFs in 24 h extracts of pure EVOH samples, cell viability was not affected (relative metabolic activity of 109.6 ± 30.7, 108.6 ± 20.5 and 109.5 ± 4.6% for cells cultured for 3 days in 24 h extracts from EVOH32, EVOH38 and EVOH44, respectively). In contrast, extracts from membranes loaded with IBU slightly reduced the cell viability after 3 days of culture to 90.7 ± 37.2, 81.8 ± 33.9 and 91.2 ± 22.0% for EVOH32, EVOH38 and EVOH44, respectively. Assessing the total cell number, similar values were observed after 3 days of culture in 24 h extracts (relative total DNA content of 117.7 ± 29.3, 127.5 ± 16.0 and 112 ± 24.0% for EVOH32, EVOH38 and EVOH44, respectively and 100.7 ± 26.3, 88.4 ± 18.8 and 94.9 ± 19.1% for IBU-loaded membrane extracts of EVOH32, EVOH38 and EVOH44, respectively). The slightly lower values with extracts from IBU-loaded membranes were unexpected, as several reports have shown no cytotoxicity of IBU for higher concentrations.^57,58^ Overall, this assay demonstrated that the scaffolds were compatible with biomedical applications and no acute toxicity was observed.

## 4 Conclusion

In this study, we report the design of a mechanically stable electrospun mesh as well as the release kinetics of IBU therefrom. We showed that a higher ethylene content within the EVOH copolymer used for electrospinning led to less IBU being released from the scaffolds due to strong hydrophobic interactions between the fibers and the drug. Furthermore, the proposed membranes showed excellent mechanical properties and cytocompatibility making them promising candidates for the development of drug-loaded nanofibrous patches for biomedical applications. Further research should investigate the release of other compounds using EVOH NFs as well as the ability of the patch to allow for the diffusion of drugs through the skin using both *in vitro* and *in vivo* approaches.

## Conflicts of interest

There are no conflicts of interest to declare.

## Supplementary Material

NA-005-D3NA00102D-s001
