# Supplementary material for: Ibuprofen-loaded electrospun poly(ethylene-co-vinyl alcohol) nanofibers for wound dressing applications
Source: Nanoscale Adv. 2023 Mar 7;5(8):2261–70. doi: 10.1039/d3na00102d (PMC10089083; doi:10.1039/d3na00102d)
Supplement: NA-005-D3NA00102D-s001 [file NA-005-D3NA00102D-s001.pdf]

Electronic Supplementary Material (ESI) for Nanoscale Advances.
This journal is © The Royal Society of Chemistry 2023

Supplementary information: Ibuprofen-loaded electrospun poly(ethylene-co-vinyl alcohol) nanofibers for wound dressing applications

Jean Schoeller[1,2], Karin Wuertz-Kozak[3], Stephen J. Ferguson[2], Markus Rottmar[4], Yvonne Elbs-Glatz[4], Jonathan Avaro[5], Michael Chung[6], and René M. Rossi[1]

[1]Empa, Swiss Federal Laboratories for Materials Science and Technology, Laboratory for Biomimetic Membranes and Textiles, 9014 St. Gallen, Switzerland
[2]ETH Zürich, Institute for Biomechanics, 8093 Zürich, Switzerland
[3]Rochester Institute of Technology (RIT), Department of Biomedical Engineering, Rochester, NY 14623, United States
[4]Empa, Swiss Federal Laboratories for Materials Science and Technology, Laboratory for Biointerfaces, 9014 St. Gallen, Switzerland
[5]Empa, Swiss Federal Laboratories for Materials Science and Technology, Center for X-ray analytics, 8600, Dübendorf, Switzerland
[6]School of Engineering, The University of Edinburgh, King's Buildings, EH9 3JL, Edinburgh, United Kingdom

March 7, 2023

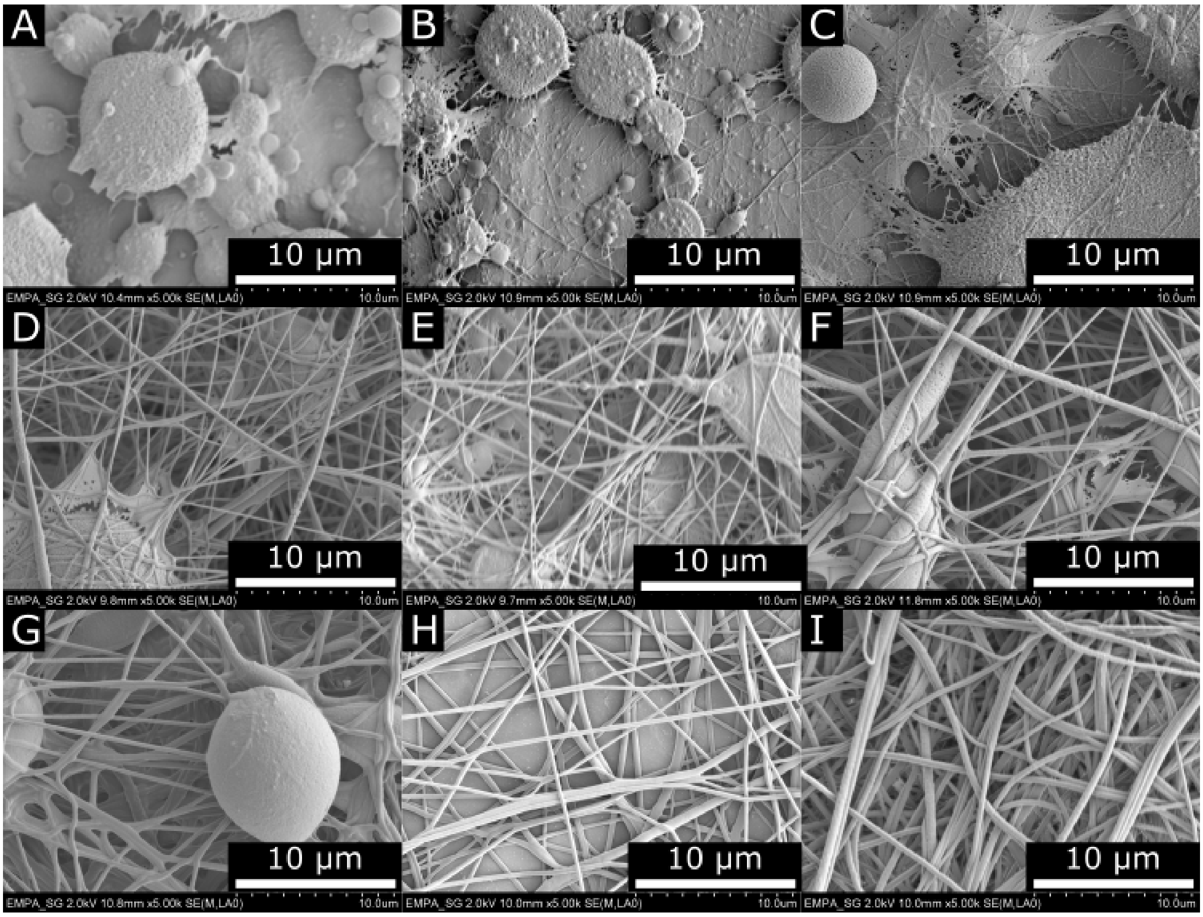

Figure S1: SEM pictures for EVOH32 electrospun nanofibers electrospun at 20 cm, 15/-5kV, 10 μL.min^{-1} and (A) 4, (B) 6, (C) 8, (D) 10, (E) 12, (F) 14, (G) 16, (H) 18 and (I) the fibers used in this study.

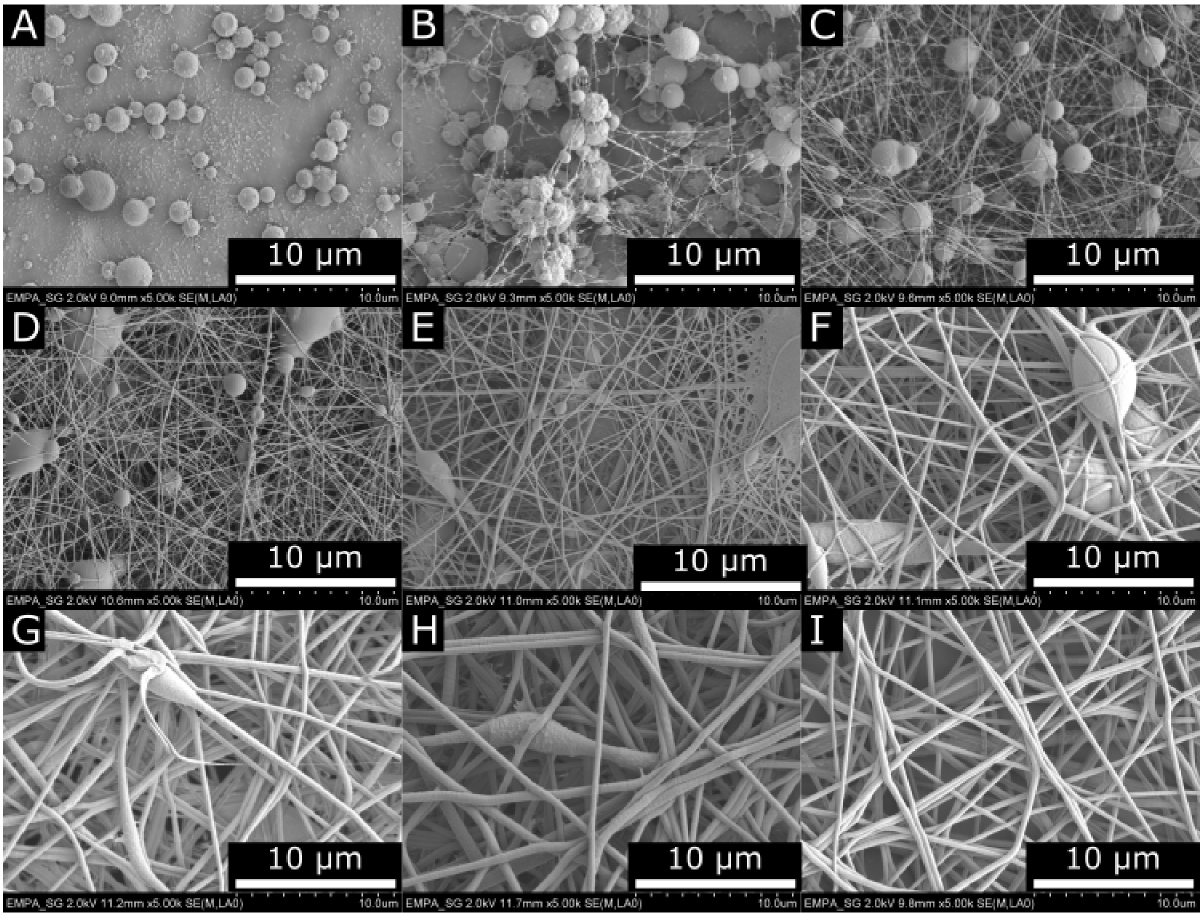

Figure S2: SEM pictures for EVOH38 electrospun nanofibers electrospun at 20 cm, 15/-5kV, 10 μ L.min^{-1} and (A) 6, (B) 8, (C) 10, (D) 12, (E) 14, (F) 16, (G) 18, (H) 20 and (I) the fibers used in this study.

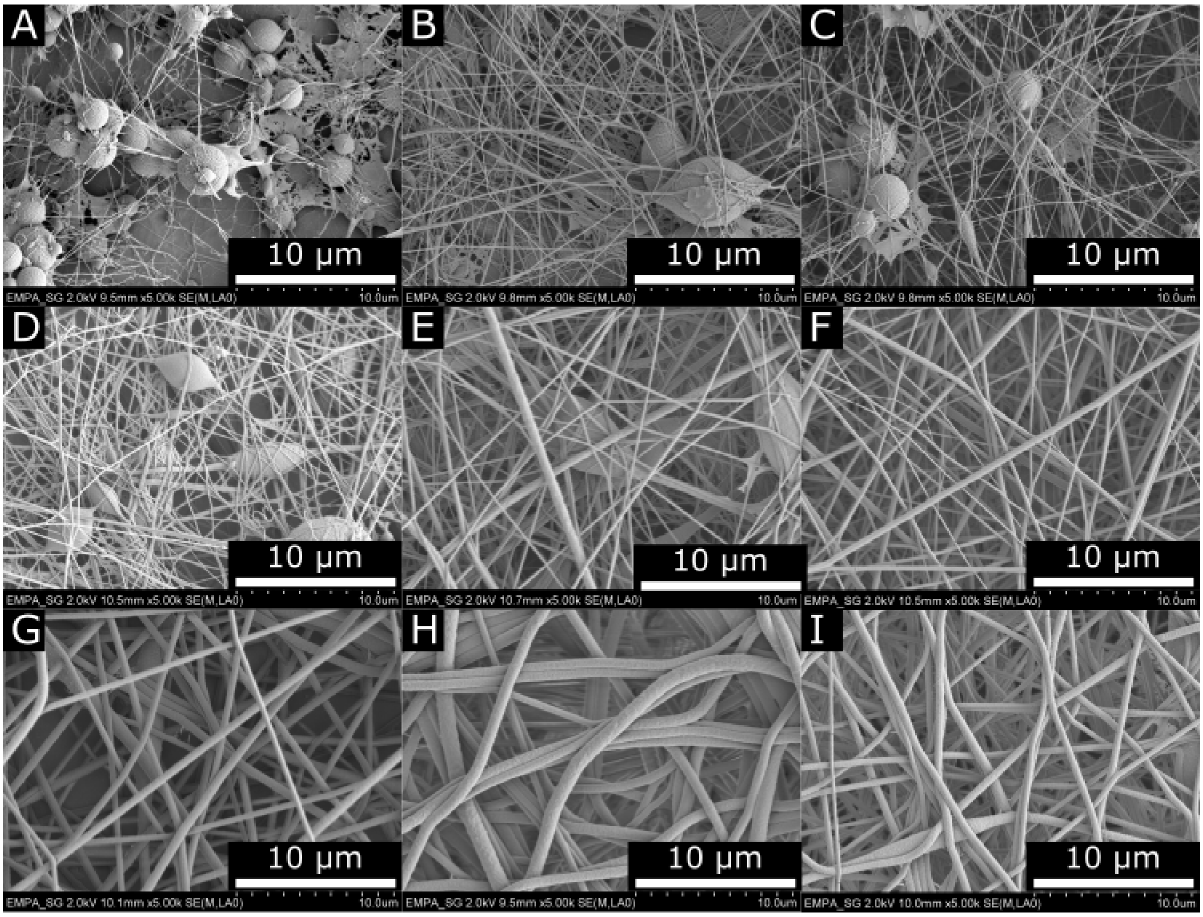

Figure S3: SEM pictures for EVOH44 electrospun nanofibers electrospun at 20 cm, 15/-5kV, 10 µL.min^{-1} and (A) 6, (B) 8, (C) 10, (D) 12 (E) 14, (F) 16, (G) 18, (H) 20 and (I) the fibers used in this study.

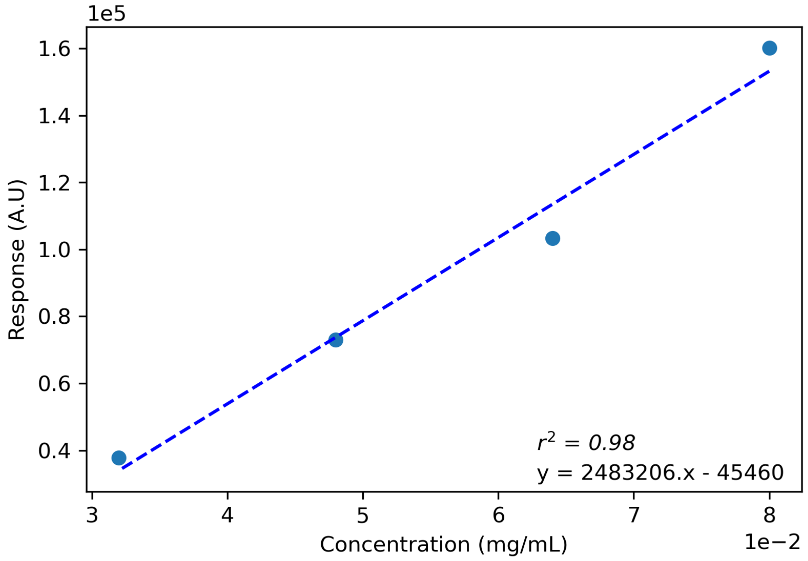

Figure S4: Calibration curve for the detection of ibuprofen in PBS.

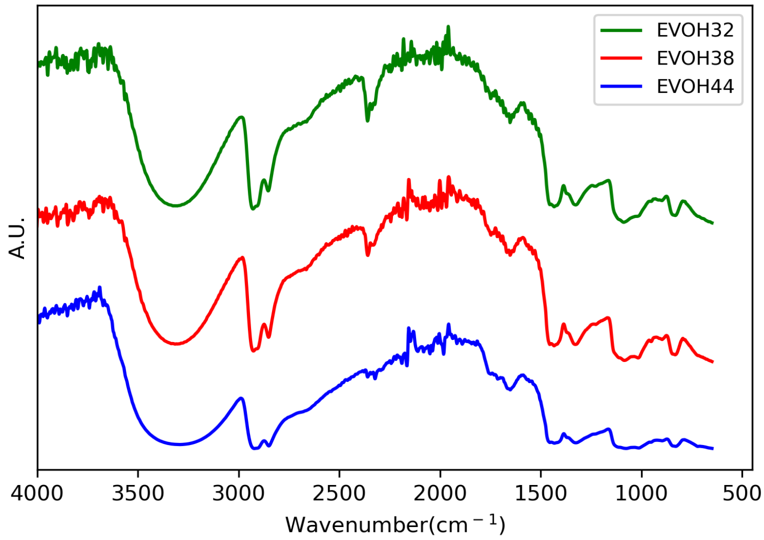

Figure S5: FTIR spectra of EVOH32, EVOH38 and EVOH44

Table S1: Apex Track integration parameters for TargetLinx method for UPLC analysis of ibuprofen

Retention time	3.38 ± 0.2 minutes
Peak-to-peak baseline noise	9772.00
Peak Width at 5% Height	0.45 minutes
Baseline Start Threshold	5.0%
Baseline End Threshold	0.0%

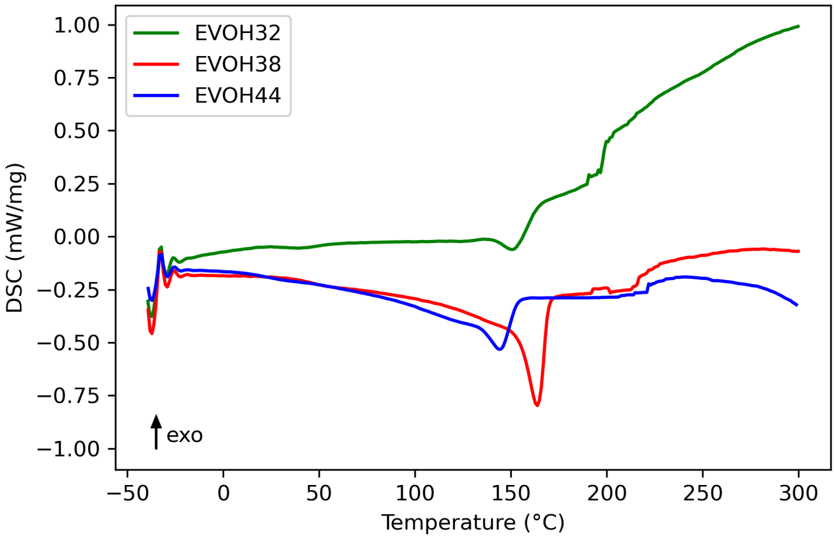

Figure S6: DSC of EVOH32, EVOH38 and EVOH44 - second heating cycle

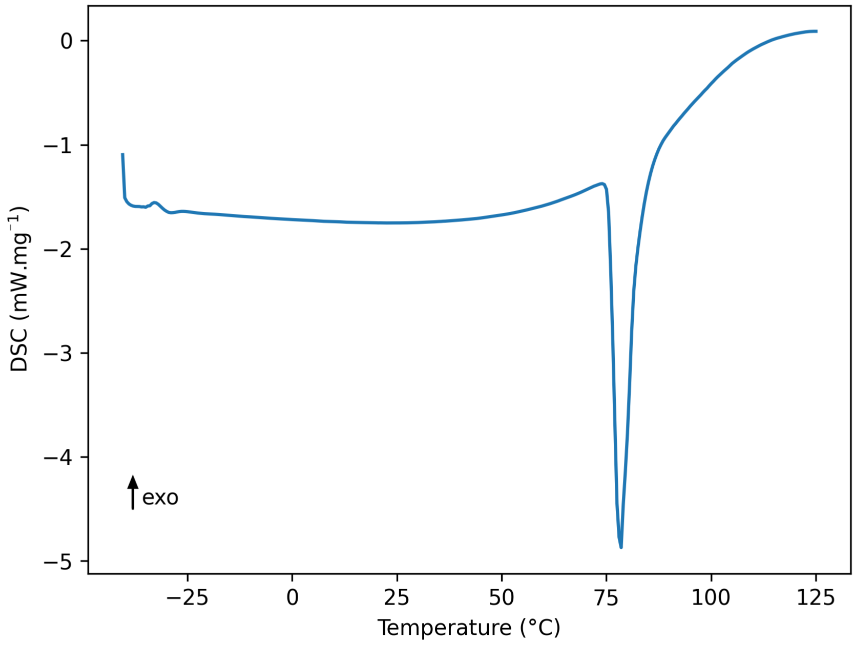

Figure S7: DSC of ibuprofen

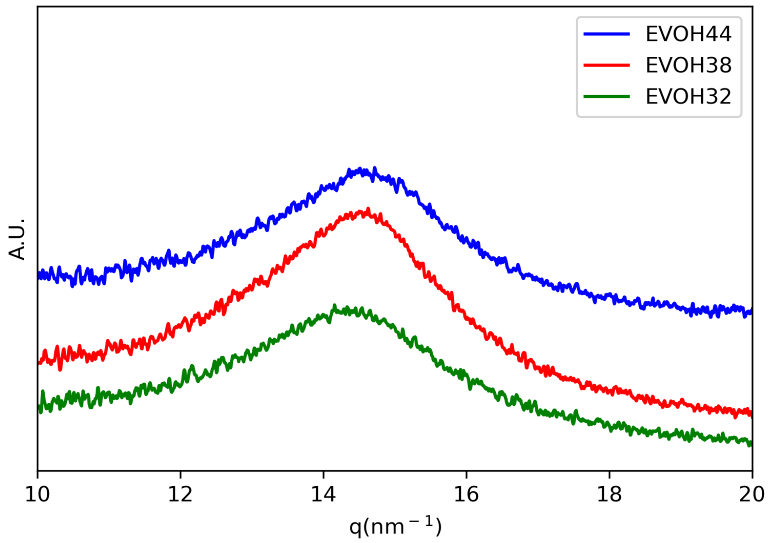

Figure S8: WAXS data of EVOH32, EVOH38 and EVOH44

Table S2: Parameters of the desorption model used to fit the drug release from EVOH32, EVOH38 and EVOH44

Membrane	Temperature	α	τ	r^2
EVOH32	25°C	0.77	0.15	0.99
	37.5°C	0.81	0.18	0.98
EVOH38	25°C	0.67	.21	0.98
	37.5°C	0.77	0.18	0.92
EVOH44	25°C	0.49	0.31	0.91
	37.5°C	0.87	0.24	0.96

Table S3: Statistical analysis for the release of IBU for EVOH32, EVOH38 and EVOH44 in function of temperature

Groups	p-value (Dunn Test)	Significance
EVOH32 25°C & 37.5°C	0.000442	**
EVOH38 25°C & 37.5°C	0.000669	**
EVOH44 25°C & 37.5°C	2.92e-13	**

Table S4: Statistical analysis for the release of IBU for EVOH32, EVOH38 and EVOH44 in function of ethylene content. (p-value of Kruskal Wallis of 3.35e-16)

Groups	p-value (Dunn Test)	Significance
EVOH32 25°C & EVOH38 25°C	1.96e-3	**
EVOH38 25°C & EVOH44 25°C	1.5e-7	**
EVOH44 25°C & EVOH32 25°C	6.86e-17	**

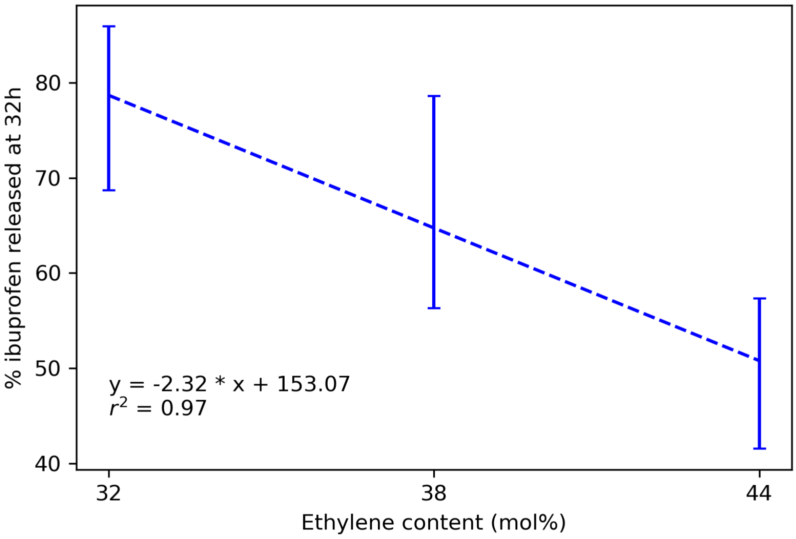

Figure S9: Amount of ibuprofen released depending on the ethylene content after 32h
